# Supplementary material for: Surrounding greenness is associated with lower risk and burden of low birth weight in Iran
Source: Nat Commun. 2023 Nov 21;14:7595. doi: 10.1038/s41467-023-43425-6 (PMC10663448; doi:10.1038/s41467-023-43425-6)
Supplement: Supplementary file 1 — Supplementary Information [file 41467_2023_43425_MOESM1_ESM.pdf]

Supplementary Information

Surrounding greenness is associated with lower risk and burden of low birth weight in Iran

Siqi Luo [1, 4], Yaqi Wang [1, 4], Fatemeh Mayvaneh [2, *], Helder Relvas [3], Mohammad Baaghideh [2], Kai Wang [1], Yang Yuan [1], Zhouxin Yin [1], Yunquan Zhang [1, *]

[1] Institute of Social Development and Health Management, Hubei Province Key Laboratory of Occupational Hazard Identification and Control, School of Public Health, Wuhan University of Science and Technology, Wuhan 430065, China

[2] Faculty of Geography and Environmental Sciences, Hakim Sabzevari University, Sabzevar 9617916487, Khorasan Razavi, Iran

[3] CESAM & Department of Environment and Planning, University of Aveiro, 3810-193 Aveiro, Portugal

[4] These authors contributed equally: Siqi Luo, Yaqi Wang.

[*] Corresponding authors:

Dr. Yunquan Zhang, School of Public Health, Wuhan University of Science and Technology, Wuhan 430065, China. Email: YunquanZhang@wust.edu.cn.

Dr. Fatemeh Mayvaneh, Faculty of Geography and Environmental Sciences, Hakim Sabzevari University, Sabzevar 9617916487, Khorasan Razavi, Iran. Email: fmayvaneh@yahoo.com.

Table of contents

Table S1 Sensitivity analyses for the associations between LBW/TLBW and greenness exposures within multiple buffers throughout the pregnancy.

Table S2 Associations between LBW/TLBW and quartiles of normalized difference vegetation index exposure within multiple buffers.

Table S3 Associations between LBW/TLBW and quartiles of enhanced vegetation index exposure within multiple buffers.

Table S4 Stratified analyses for the associations of LBW and TLBW with greenness exposure within a 3000-m buffer.

Table S5 Estimates of greenness-related attributable fractions and avoidable numbers of LBW and TLBW by achieving targets of mean NDVI or EVI.

Figure S1 Exposure-response associations between LBW/TLBW and NDVI (a) or EVI (b) within multiple buffers.

Figure S2 Subgroup-specific OR estimates of LBW and TLBW associated with a 0.1-unit increase in NDVI and EVI within a 500-m buffer.

Figure S3 Subgroup-specific OR estimates of LBW and TLBW associated with a 0.1-unit increase in NDVI and EVI within a 1000-m buffer.

Figure S4 Subgroup-specific OR estimates of LBW and TLBW associated with a 0.1-unit increase in NDVI and EVI within a 2000-m buffer.

Figure S5 Geographic locations of 749 hospitals across Iran included in this study.

Table S1 Sensitivity analyses for the associations between LBW/TLBW and greenness exposures within multiple buffers throughout the pregnancy.

Analytic strategies	Buffer	LBW (OR, 95% CI)		TLBW (OR, 95% CI)	
		NDVI	EVI	NDVI	EVI
**Including neonates with birth weights between 500 g and 5000 g (n = 4,015,616)**					
	**500 m**	0.926 (0.923–0.929)	0.908 (0.905–0.912)	0.915 (0.912–0.919)	0.895 (0.890–0.899)
	**1000 m**	0.920 (0.917–0.922)	0.898 (0.895–0.902)	0.908 (0.904–0.911)	0.884 (0.879–0.888)
	**2000 m**	0.912 (0.909–0.915)	0.887 (0.883–0.891)	0.899 (0.896–0.903)	0.872 (0.867–0.876)
	**3000 m**	0.907 (0.904–0.910)	0.879 (0.875–0.883)	0.893 (0.889–0.897)	0.862 (0.857–0.867)
**Incorporating singleton live births (n = 3,976,387)**					
	**500 m**	0.929 (0.926–0.932)	0.912 (0.909–0.916)	0.919 (0.916–0.922)	0.900 (0.896–0.904)
	**1000 m**	0.922 (0.920–0.925)	0.903 (0.899–0.906)	0.911 (0.908–0.914)	0.889 (0.884–0.893)
	**2000 m**	0.916 (0.913–0.919)	0.892 (0.888–0.896)	0.903 (0.900–0.907)	0.878 (0.873–0.882)
	**3000 m**	0.910 (0.907–0.913)	0.884 (0.880–0.888)	0.897 (0.893–0.900)	0.868 (0.863–0.873)
**Restricting maternal age at 13–50 years old (n = 3,949,357)**					
	**500 m**	0.929 (0.927–0.932)	0.913 (0.909–0.916)	0.920 (0.917–0.924)	0.902 (0.897–0.906)
	**1000 m**	0.923 (0.920–0.926)	0.903 (0.900–0.907)	0.913 (0.909–0.916)	0.891 (0.886–0.895)
	**2000 m**	0.916 (0.913–0.919)	0.893 (0.889–0.897)	0.905 (0.902–0.909)	0.880 (0.875–0.884)
	**3000 m**	0.911 (0.908–0.914)	0.885 (0.881–0.889)	0.899 (0.895–0.902)	0.870 (0.865–0.875)

(Table S1 continued)

Analytic strategies	Buffer	LBW (OR, 95% CI)		TLBW (OR, 95% CI)	
		NDVI	EVI	NDVI	EVI
**Including neonates whose mothers have no diabetes and hypotension (n = 3,862,524)**					
	**500 m**	0.928 (0.925–0.931)	0.911 (0.908–0.915)	0.919 (0.916–0.923)	0.901 (0.896–0.905)
	**1000 m**	0.922 (0.919–0.925)	0.902 (0.898–0.906)	0.912 (0.908–0.915)	0.890 (0.885–0.894)
	**2000 m**	0.915 (0.912–0.918)	0.891 (0.888–0.895)	0.904 (0.901–0.908)	0.879 (0.874–0.883)
	**3000 m**	0.909 (0.906–0.912)	0.883 (0.879–0.887)	0.897 (0.894–0.901)	0.869 (0.864–0.873)
**Incorporating neonates whose mothers have no delivery complications (n = 3,914,965)**					
	**500 m**	0.929 (0.926–0.932)	0.913 (0.909–0.916)	0.920 (0.917–0.924)	0.902 (0.898–0.906)
	**1000 m**	0.923 (0.920–0.925)	0.903 (0.899–0.907)	0.913 (0.909–0.916)	0.891 (0.887–0.896)
	**2000 m**	0.916 (0.913–0.919)	0.893 (0.889–0.896)	0.905 (0.902–0.909)	0.880 (0.876–0.885)
	**3000 m**	0.910 (0.907–0.913)	0.884 (0.880–0.888)	0.899 (0.895–0.902)	0.870 (0.866–0.875)
**Additionally adjusting for provincial-level SES variables (n = 4,021,741)**					
	**500 m**	0.938 (0.935–0.941)	0.926 (0.922–0.930)	0.928 (0.925–0.932)	0.914 (0.910–0.919)
	**1000 m**	0.932 (0.929–0.935)	0.918 (0.914–0.921)	0.921 (0.917–0.925)	0.904 (0.900–0.909)
	**2000 m**	0.925 (0.922–0.928)	0.907 (0.903–0.911)	0.913 (0.909–0.917)	0.893 (0.888–0.898)
	**3000 m**	0.919 (0.916–0.922)	0.899 (0.895–0.904)	0.906 (0.903–0.910)	0.884 (0.879–0.889)

Abbreviations: LBW, low birth weight; TLBW, term low birth weight; OR, odds ratio; CI, confidence interval; NDVI, normalized difference vegetation index; EVI, enhanced vegetation index; SES, socioeconomic status.

Table S2 Associations between LBW/TLBW and quartiles of normalized difference vegetation index exposure within multiple buffers.

Buffer	Quantiles	Median	LBW		TLBW	
			OR (95% CI)	*P*-trend*	OR (95% CI)	*P*-trend*
**500 m**	Q1	0.103	1 (Ref.)	<0.001	1 (Ref.)	<0.001
	Q2	0.219	0.981 (0.979–0.982)		0.975 (0.974–0.977)	
	Q3	0.325	0.973 (0.972–0.975)		0.967 (0.966–0.969)	
	Q4	0.554	0.965 (0.963–0.966)		0.961 (0.960–0.963)	
**1000 m**	Q1	0.106	1 (Ref.)	<0.001	1 (Ref.)	<0.001
	Q2	0.221	0.984 (0.983–0.985)		0.978 (0.977–0.980)	
	Q3	0.332	0.973 (0.971–0.974)		0.966 (0.964–0.967)	
	Q4	0.541	0.962 (0.960–0.963)		0.958 (0.957–0.960)	
**2000 m**	Q1	0.109	1 (Ref.)	<0.001	1 (Ref.)	<0.001
	Q2	0.233	0.982 (0.980–0.983)		0.976 (0.974–0.978)	
	Q3	0.336	0.970 (0.969–0.972)		0.963 (0.961–0.965)	
	Q4	0.524	0.960 (0.959–0.962)		0.956 (0.955–0.958)	

These estimates are derived from the fully-adjusted model, where covariates include maternal demographic characteristics, fetal variables, and environmental factors. Error bars represent 95% confidence intervals. The statistical tests are two-sided. Abbreviations: LBW, low birth weight; TLBW, term low birth weight; OR, odds ratio; CI, confidence interval.

* Tested by introducing the median value of each quartile as a continuous variable in the logistic regression model.

Table S3 Associations between LBW/TLBW and quartiles of enhanced vegetation index exposure within multiple buffers.

Buffer	Quantile	Median	LBW		TLBW	
			OR (95% CI)	*P*-trend*	OR (95% CI)	*P*-trend*
**500 m**	Q1	0.070	1 (Ref.)	<0.001	1 (Ref.)	<0.001
	Q2	0.154	0.983 (0.981–0.984)		0.976 (0.975–0.978)	
	Q3	0.230	0.978 (0.976–0.979)		0.971 (0.969–0.973)	
	Q4	0.418	0.965 (0.964–0.967)		0.962 (0.960–0.964)	
**1000 m**	Q1	0.071	1 (Ref.)	<0.001	1 (Ref.)	<0.001
	Q2	0.160	0.986 (0.985–0.988)		0.980 (0.978–0.981)	
	Q3	0.235	0.974 (0.972–0.975)		0.966 (0.965–0.968)	
	Q4	0.410	0.964 (0.962–0.965)		0.961 (0.959–0.963)	
**2000 m**	Q1	0.072	1 (Ref.)	<0.001	1 (Ref.)	<0.001
	Q2	0.168	0.982 (0.981–0.984)		0.976 (0.975–0.978)	
	Q3	0.239	0.970 (0.969–0.972)		0.963 (0.962–0.965)	
	Q4	0.390	0.963 (0.962–0.964)		0.960 (0.958–0.961)	

These estimates are derived from the fully-adjusted model, where covariates include maternal demographic characteristics, fetal variables, and environmental factors. Error bars represent 95% confidence intervals. The statistical tests are two-sided. Abbreviations: LBW, low birth weight; TLBW, term low birth weight; OR, odds ratio; CI, confidence interval.

* Tested by introducing the median value of each quartile as a continuous variable in the logistic regression model.

Table S4 Stratified analyses for the associations of LBW and TLBW with greenness exposure within a 3000-m buffer.

Subgroup	NDVI		EVI	
	OR (95% CI)	*P-value**	OR (95% CI)	*P-value**
**LBW**				
**Age**				
≤25	0.895 (0.890–0.900)	Ref.	0.862 (0.856–0.868)	Ref.
(25, 35]	0.922 (0.918–0.926)	<0.001	0.901 (0.895–0.907)	<0.001
>35	0.923 (0.914–0.932)	<0.001	0.903 (0.891–0.914)	<0.001
**Education level**				
Below high school	0.897 (0.893–0.901)	Ref.	0.864 (0.859–0.870)	Ref.
High school	0.936 (0.930–0.943)	<0.001	0.920 (0.911–0.928)	<0.001
College or above	0.940 (0.933–0.948)	<0.001	0.925 (0.915–0.935)	<0.001
**Residence**				
City	0.919 (0.915–0.923)	Ref.	0.896 (0.892–0.901)	Ref.
Village	0.893 (0.887–0.898)	<0.001	0.860 (0.853–0.867)	<0.001
**TLBW**				
**Age**				
≤25	0.884 (0.878–0.889)	Ref.	0.848 (0.841–0.856)	Ref.
(25, 35]	0.910 (0.905–0.916)	<0.001	0.887 (0.881–0.894)	<0.001
>35	0.913 (0.902–0.924)	<0.001	0.888 (0.874–0.902)	<0.001
**Education level**				
Below high school	0.883 (0.878–0.888)		0.846 (0.840–0.852)	Ref.
High school	0.931 (0.924–0.939)	<0.001	0.914 (0.904–0.924)	<0.001
College or above	0.935 (0.926–0.944)	<0.001	0.922 (0.911–0.934)	<0.001
**Residence**				
City	0.908 (0.904–0.912)	Ref.	0.884 (0.878–0.890)	Ref.
Village	0.879 (0.873–0.886)	<0.001	0.842 (0.833–0.850)	<0.001

These estimates are derived from the fully-adjusted model, where covariates include maternal demographic characteristics, fetal variables, and environmental factors. Error bars represent 95% confidence intervals. * refers to effect heterogeneity between subgroups, which is examined through a fixed effect meta-regression method. The statistical tests are two-sided. Abbreviations: OR, odds ratio; CI, confidence interval; NDVI, normalized difference vegetation index; EVI, enhanced vegetation index; LBW, low birth weight; TLBW, term low birth weight.

Table S5 Estimates of greenness-related attributable fraction and avoidable number of LBW and TLBW by achieving targets of mean NDVI or EVI.

Exposure	Attributable fraction (%, 95% CI)		Avoidable number (n, 95% CI)	
	LBW	TLBW	LBW	TLBW
**NDVI**				
**NDVI$_{-500m}$**	4.7 (4.4–4.9)	5.9 (5.6–6.2)	4233 (4016–4451)	2292 (2180–2405)
**NDVI$_{-1000m}$**	5.1 (4.9–5.4)	6.4 (6.1–6.7)	4637 (4413–4863)	2473 (2358–2589)
**NDVI$_{-2000m}$**	5.5 (5.2–5.7)	6.9 (6.6–7.2)	4956 (4721–5192)	2682 (2562–2802)
**NDVI$_{-3000m}$**	5.6 (5.4–5.9)	6.9 (6.6–7.3)	5099 (4855–5343)	2697 (2573–2822)
**EVI**				
**EVI$_{-500m}$**	4.4 (4.1–4.6)	5.6 (5.3–5.9)	3931 (3711–4152)	2173 (2060–2287)
**EVI$_{-1000m}$**	4.8 (4.6–5.1)	6.1 (5.8–6.4)	4374 (4147–4601)	2361 (2244–2479)
**EVI$_{-2000m}$**	5.2 (4.9–5.5)	6.6 (6.3–7.0)	4702 (4462–4942)	2584 (2461–2707)
**EVI$_{-3000m}$**	5.3 (5.0–5.6)	6.6 (6.3–7.0)	4807 (4556–5059)	2584 (2455–2712)

Abbreviations: LBW, low birth weight; TLBW, term low birth weight; CI, confidence interval; NDVI, normalized difference vegetation index; EVI, enhanced vegetation index.

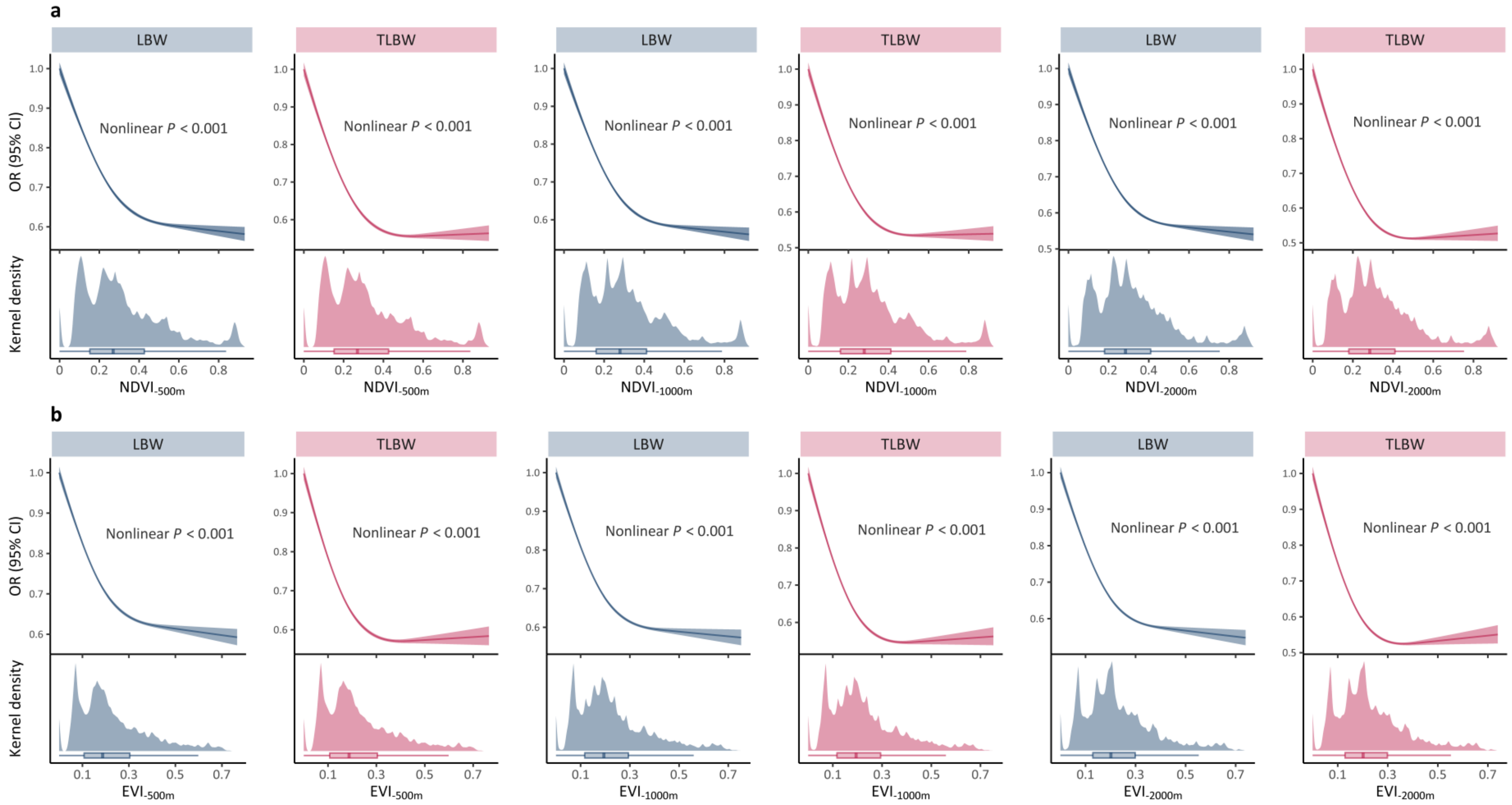

Figure S1 Exposure-response associations between LBW/TLBW and NDVI (a) or EVI (b) within multiple buffers. The curve was modeled using restricted cubic splines function with 3 knots placed at the 10th, 50th, and 90th percentiles of greenness exposure distribution. Abbreviations: OR, odds ratio; CI, confidence interval; LBW, low birth weight; TLBW, term low birth weight; NDVI, normalized difference vegetation index; EVI, enhanced vegetation index.

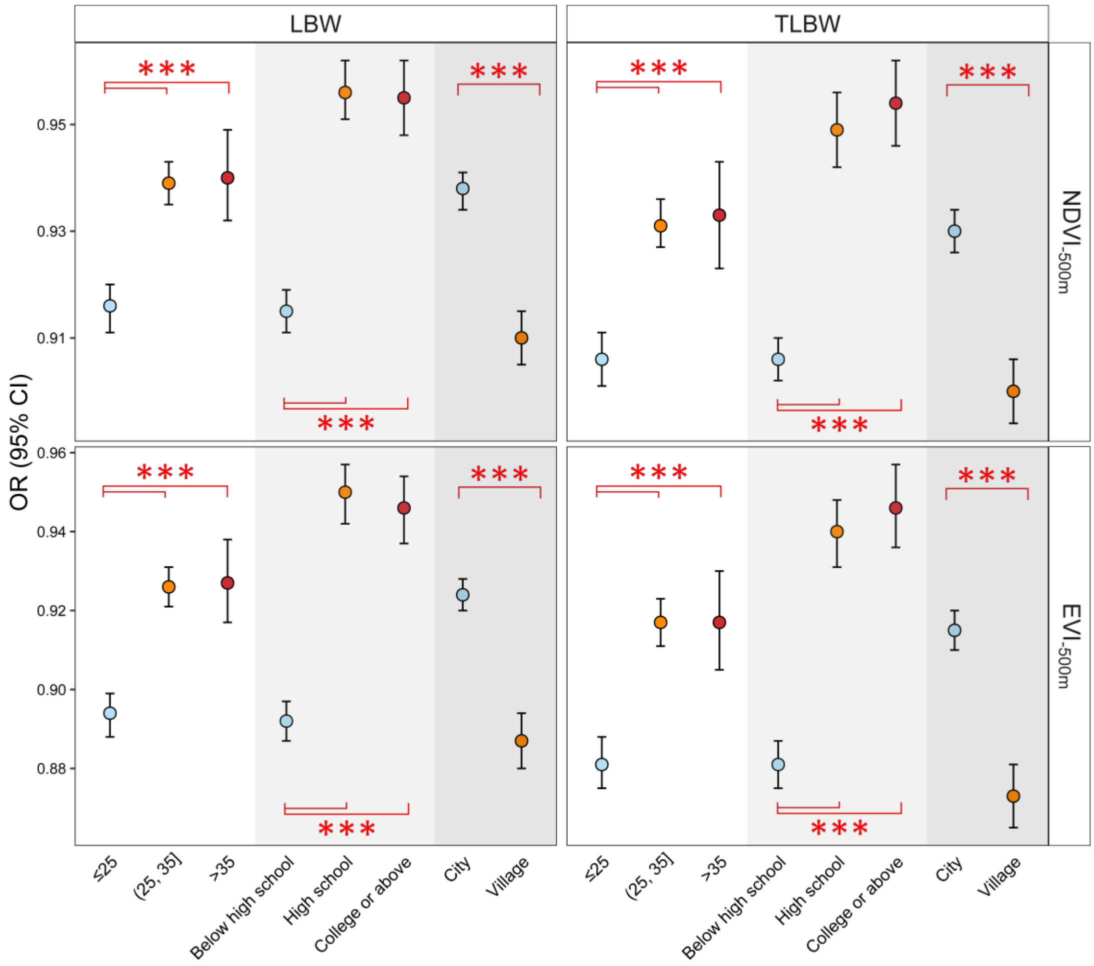

Figure S2 Subgroup-specific OR estimates of LBW and TLBW associated with a 0.1-unit increase in NDVI and EVI within a 500-m buffer. These estimates are derived from the fully-adjusted model, where covariates include maternal demographic characteristics, fetal variables, and environmental factors. Error bars represent 95% confidence intervals. Effect heterogeneity between subgroups is examined through a fixed effect meta-regression method, and the statistical tests are two-sided. The exact sample sizes for subgroups are exhibited in Table 1. *** indicates *P* <0.001 for effect heterogeneity between subgroups. Abbreviations: OR, odds ratio; LBW, low birth weight; TLBW, term low birth weight; NDVI, normalized difference vegetation index; EVI, enhanced vegetation index.

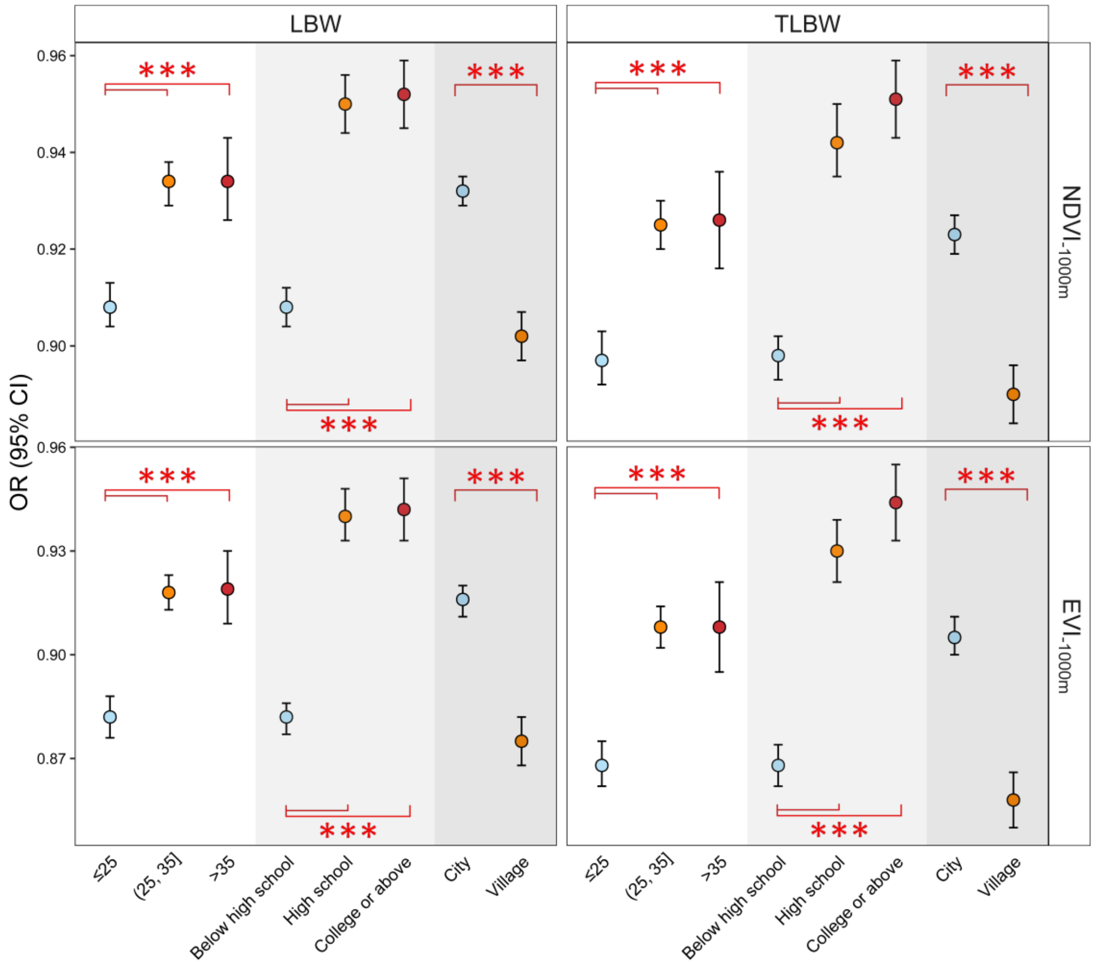

Figure S3 Subgroup-specific OR estimates of LBW and TLBW associated with a 0.1-unit increase in NDVI and EVI within a 1000-m buffer. These estimates are derived from the fully-adjusted model, where covariates include maternal demographic characteristics, fetal variables, and environmental factors. Error bars represent 95% confidence intervals. Effect heterogeneity between subgroups is examined through a fixed effect meta-regression method, and the statistical tests are two-sided. The exact sample sizes for subgroups are exhibited in Table 1. *** indicates *P* <0.001 for effect heterogeneity between subgroups. Abbreviations: OR, odds ratio; LBW, low birth weight; TLBW, term low birth weight; NDVI, normalized difference vegetation index; EVI, enhanced vegetation index.

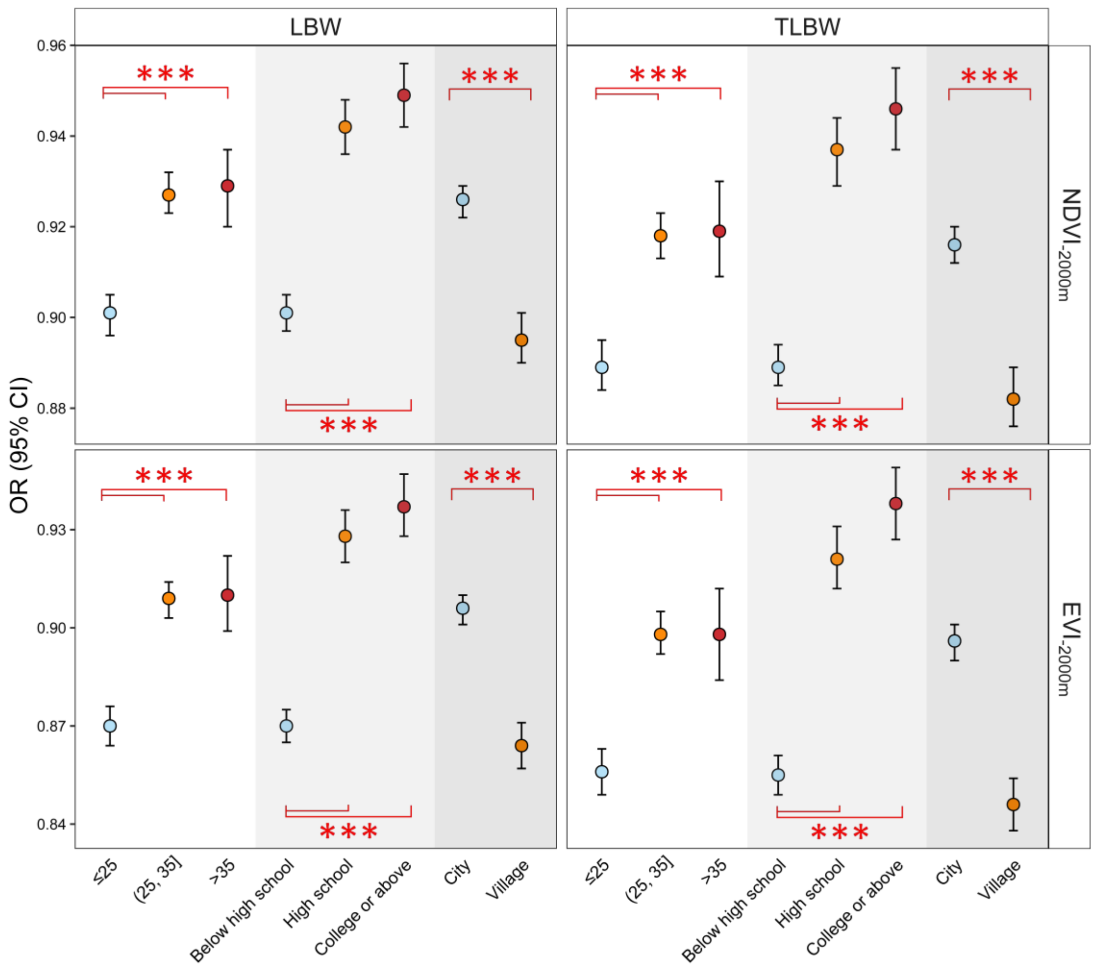

Figure S4 Subgroup-specific OR estimates of LBW and TLBW associated with a 0.1-unit increase in NDVI and EVI within a 2000-m buffer. These estimates are derived from the fully-adjusted model, where covariates include maternal demographic characteristics, fetal variables, and environmental factors. Error bars represent 95% confidence intervals. Effect heterogeneity between subgroups is examined through a fixed effect meta-regression method, and the statistical tests are two-sided. The exact sample sizes for subgroups are exhibited in Table 1. *** indicates *P* <0.001 for effect heterogeneity between subgroups. Abbreviations: OR, odds ratio; LBW, low birth weight; TLBW, term low birth weight; NDVI, normalized difference vegetation index; EVI, enhanced vegetation index.

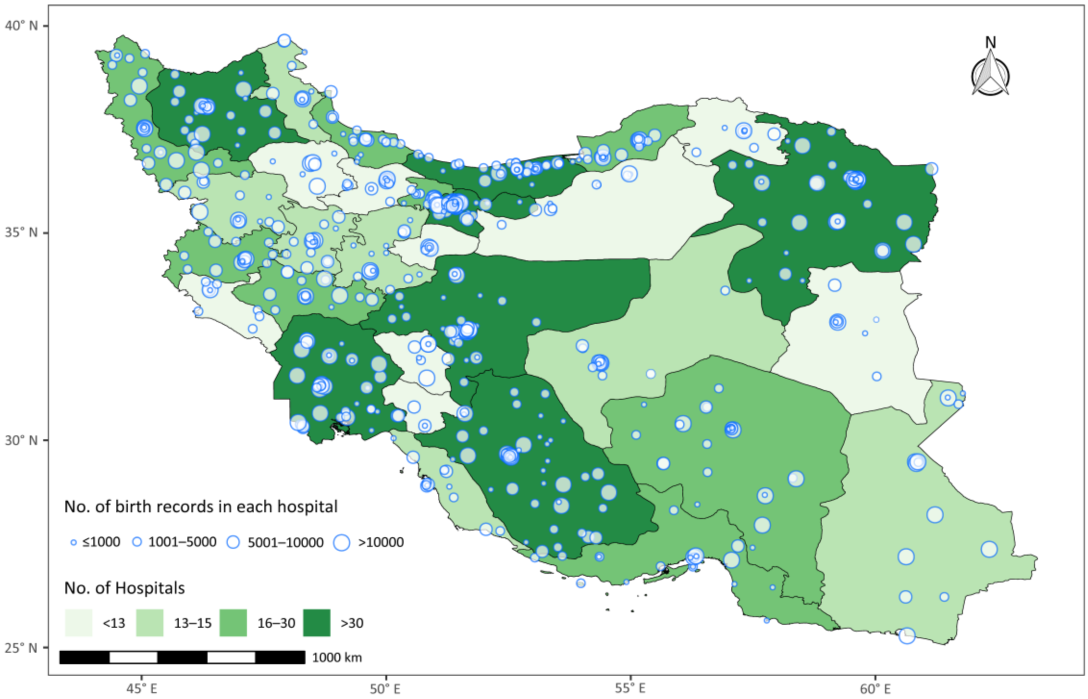

Figure S5 Geographic locations of 749 hospitals across Iran included in this study. Shapefile for Iran is publicly downloaded from https://gadm.org/download_country.html.
