# Supplementary material for: Surrounding greenness is associated with lower risk and burden of low birth weight in Iran
Source: Nat Commun. 2023 Nov 21;14:7595. doi: 10.1038/s41467-023-43425-6 (PMC10663448; doi:10.1038/s41467-023-43425-6)
Supplement: Supplementary file 3 — Description of Additional Supplementary Files [file 41467_2023_43425_MOESM3_ESM.pdf]

Description of Additional Supplementary Files

File name: Supplementary Code

Description: R codes for statistical analyses and drawing figures.
