# Supplementary material for: Surrounding greenness is associated with lower risk and burden of low birth weight in Iran
Source: Nat Commun. 2023 Nov 21;14:7595. doi: 10.1038/s41467-023-43425-6 (PMC10663448; doi:10.1038/s41467-023-43425-6)
Supplement: Supplementary file 4 — Supplementary Code [file 41467_2023_43425_MOESM4_ESM.pdf]

Supplementary R Codes

Surrounding greenness is associated with lower risk and burden of low birth weight in Iran

Siqi Luo, Yaqi Wang, Fatemeh Mayvaneh, Helder Relvas, Mohammad Baaghideh, Kai Wang, Yang Yuan, Zhouxin Yin, Yunquan Zhang

Contents

1. Codes for the regression models in the main text 2

2. Codes for Figure 2 4

3. Codes for Figure 3 6

4. Codes for Figure 4 10

5. Codes for Figure 5 12

1. Codes for the regression models in the main text

```
##Codes are not utilized for running, but to aid interpretation of the statistical models used in
the study.
## load the packages
library(xlsx);library(dplyr)
##load the analyzed data
load("data_iran.rdata")
## Construct a function to extract the result
ect <- function(model){
  beta <- summary(model)$coef[2,"Estimate"]
  low <- (summary(model)$coef[2,"Estimate"] - 1.96*summary(model)$coef[2, "Std. Error"])
  high <- (summary(model)$coef[2,"Estimate"] + 1.96*summary(model)$coef[2, "Std.
Error"])
  p   <- summary(model)$coef[2,"Pr(>|z|)"]
  table <- cbind(beta,low,high,p)
  return(table)}
outcomes<-list(data$lbw,data$tlbw)
exposures<-list(data$NDVI500,data$NDVI1000,data$NDVI2000,
                data$NDVI3000,data$EVI500,data$EVI1000,
                data$EVI2000,data$EVI3000)
################################
########### model 1 ############
################################
mod1<-lapply(outcomes,function(y){
  a<-lapply(exposures,function(x){
    model <- glm(y~x,
                 family=binomial(link = "logit"),
                 data=data)
    d<-ect(model)
    return(d)
  })
})
rbind(mod1[[1]],mod1[[2]]) %>%
  do.call("rbind",.) %>%
  as.data.frame() %>%
  mutate(ci=paste0(round(exp(beta)^0.1,3),
                   " [",round(exp(low)^0.1,3)," to ", round(exp(high)^0.1,3),"]")) %>%
  mutate(ci0=paste(ci,cut(p,c(-0.1,0.001,0.01,0.05,1),c("***","**","*","")),sep=" "),
         exposure=rep(c("NDVI500","NDVI1000","NDVI2000","NDVI3000",
                        "EVI500","EVI1000","EVI2000","EVI3000"),each=2),
         outcome=c(rep(c("lbw","tlbw"),8)),
         mod="Model 1")->model1
```

```
################################
########### model 2 ############
################################
mod2<-lapply(outcomes,function(y){
  a<-lapply(exposures,function(x){
    model <- glm(y~x+age+Sex+gw,
                 family=binomial(link = "logit"),
                 data=data)
    d<-ect(model)
    return(d)
  })
})
rbind(mod2[[1]],mod2[[2]]) %>%
  do.call("rbind",.) %>%
  as.data.frame() %>%
  mutate(ci=paste0(round(exp(beta)^0.1,3),
                   " [",round(exp(low)^0.1,3)," to ", round(exp(high)^0.1,3),"]")) %>%
  mutate(ci0=paste(ci,cut(p,c(-0.1,0.001,0.01,0.05,1),c("***","**","*","")),sep=" "),
         exposure=rep(c("NDVI500","NDVI1000","NDVI2000","NDVI3000",
                        "EVI500","EVI1000","EVI2000","EVI3000"),each=2),
         outcome=c(rep(c("lbw","tlbw"),8)),
         mod="Model 2")->model2
################################
########### model 3 ############
################################
mod3<-lapply(outcomes,function(y){
  a<-lapply(exposures,function(x){
    model <- glm(y~x+age+
                   Sex+order_cat+parity_cat+
                   season+nationality+residence+
                   deli_type+education+gw+hospital,
                 family=binomial(link = "logit"),
                 data=data)
    d<-ect(model)
    return(d)
  })
})
rbind(mod3[[1]],mod3[[2]]) %>%
  do.call("rbind",.) %>%
  as.data.frame() %>%
  mutate(ci=paste0(round(exp(beta)^0.1,3),
                   " [",round(exp(low)^0.1,3)," to ", round(exp(high)^0.1,3),"]")) %>%
```

```
mutate(ci0=paste(ci,cut(p,c(-0.1,0.001,0.01,0.05,1),c("***","**","*","")),sep=" "),
              exposure=rep(c("NDVI500","NDVI1000","NDVI2000","NDVI3000",
                                "EVI500","EVI1000","EVI2000","EVI3000"),each=2),
              outcome=c(rep(c("lbw","tlbw"),8)),
              mod="Model 3")->model3
################################
########### model 4 ############
################################
mod4<-lapply(outcomes,function(y){
  a<-lapply(exposures,function(x){
    model <- glm(y~x+pm_preg+age+
                   splines::ns(tmean_preg,df=3)+
                   splines::ns(rh_preg,3)+
                   Sex+order_cat+parity_cat+
                   season+nationality+residence+
                   deli_type+education+gw+hospital,
                 family=binomial(link = "logit"),
                 data=data)
    d<-ect(model)
    return(d)
  })
})
rbind(mod4[[1]],mod4[[2]]) %>%
  do.call("rbind",.) %>%
  as.data.frame() %>%
  mutate(ci=paste0(round(exp(beta)^0.1,3),
                   " [",round(exp(low)^0.1,3)," to ", round(exp(high)^0.1,3),"]")) %>%
  mutate(ci0=paste(ci,cut(p,c(-0.1,0.001,0.01,0.05,1),c("***","**","*","")),sep=" "),
              exposure=rep(c("NDVI500","NDVI1000","NDVI2000","NDVI3000",
                                "EVI500","EVI1000","EVI2000","EVI3000"),each=2),
              outcome=c(rep(c("lbw","tlbw"),8)),
              mod="Model 4")->model4
```

2. Codes for Figure 2

```
##load packages
library(xlsx); library(ggplot2)
## read data
mr_res<-read.xlsx("Results.xlsx",sheetIndex = 1)
mr_res$ci<-paste0(format(round(exp(mr_res$beta*0.1),3),3),
                  " (",format(round(exp(mr_res$low*0.1),3),3),
                  "–",format(round(exp(mr_res$high*0.1),3),3),")")
```

```
mytheme<-theme(axis.text.x=element_text(size=14,color="black"),
               axis.text.y=element_text(size=14,color="black",hjust = 0),
               legend.text=element_text(size=14,color="black"),
               legend.title=element_text(size=14,color="black"),
               plot.title = element_text(size = 16,color = "black",face = "bold"),
               panel.background=element_blank(),
               panel.grid.minor=element_blank(),
               panel.grid.major=element_blank(),
               panel.border=element_blank(),
               axis.line=element_blank(),
               axis.ticks=element_blank(),
               legend.position="top",
               legend.direction = "horizontal",
               legend.key.height = unit(0.6,"cm"),
               legend.key.width = unit(1.8,"cm"),
               plot.margin = margin(-0.1,0,-0.2,0,unit = "cm"))
p1<-ggplot(data=subset(mr_res,outcome=="lbw"),aes(mod,exposure,fill=or))+
  geom_tile(color="#BD3C29",size=0.4)+
  scale_x_discrete(limits=c("Model 1","Model 2",
                            "Model 3","Model 4"),
                   labels=c("Model 1","Model 2",
                            "Model 3","Model 4"))+
  scale_y_discrete(limits=c("EVI3000","EVI2000","EVI1000","EVI500",
                            "NDVI3000","NDVI2000","NDVI1000","NDVI500"),
                   labels=c(expression(EVI["-3000m"]),
                            expression(EVI["-2000m"]),
                            expression(EVI["-1000m"]),
                            expression(EVI["-500m"]),
                            expression(NDVI["-3000m"]),
                            expression(NDVI["-2000m"]),
                            expression(NDVI["-1000m"]),
                            expression(NDVI["-500m"])))+
  scale_fill_gradient2(limits=c(0.845,0.935),name="OR",
                       breaks=c(0.85,0.87,0.89,0.91,0.93),
                       labels=c(0.85,0.87,0.89,0.91,0.93),
                       low = "#2c7bb6",mid="#ffffbf",high="#d7191c",midpoint = 0.89)+
  labs(x="",y="",title = "a")+
  geom_text(aes(label=ci),size=5,hjust=0.5)+mytheme+
  theme(axis.text.x = element_blank())
p2<-ggplot(data=subset(mr_res,outcome=="tlbw"),aes(mod,exposure,fill=or))+
  geom_tile(color="#BD3C29",size=0.4)+
```

```
  scale_x_discrete(limits=c("Model 1","Model 2",
                            "Model 3","Model 4"),
                   labels=c("Model 1","Model 2",
                            "Model 3","Model 4"))+
  scale_y_discrete(limits=c("EVI3000","EVI2000","EVI1000","EVI500",
                            "NDVI3000","NDVI2000","NDVI1000","NDVI500"),
                   labels=c(expression(EVI["-3000m"]),
                            expression(EVI["-2000m"]),
                            expression(EVI["-1000m"]),
                            expression(EVI["-500m"]),
                            expression(NDVI["-3000m"]),
                            expression(NDVI["-2000m"]),
                            expression(NDVI["-1000m"]),
                            expression(NDVI["-500m"])))+
  scale_fill_gradient2(limits=c(0.845,0.935),name="OR",
                       breaks=c(0.85,0.87,0.89,0.91,0.93),
                       labels=c(0.85,0.87,0.89,0.91,0.93),
                       low = "#2c7bb6",mid="#ffffbf",high="#d7191c",midpoint = 0.89)+
  labs(x="",y="",title = "b")+
  geom_text(aes(label=ci),size=5,hjust=0.5)+mytheme
library(ggpubr)
p<-ggarrange(p1,p2,nrow = 2,align = "h",
             common.legend = T,legend = "top")
cairo_pdf("figure2.pdf",width=10,height=8,family = "Calibri")
p
dev.off()
```

3. Codes for Figure 3

```
##load and read data
load("data_iran.rdata")
cr<-read.xlsx("Results.xlsx",sheetIndex = 2)
library(ggplot2)
theme0<-theme(panel.grid.major = element_blank(),
              panel.grid.minor = element_blank(),
              panel.background = element_blank(),
              axis.text.x = element_text(size = 10),
              axis.text.y = element_text(size = 8),
              axis.title = element_text(size = 12),
              strip.text.x = element_text(size = 12),
              strip.text.y = element_text(),
              strip.background = element_rect( fill="white",colour = "black"))
```

```
########NDVI+LBW
ggplot(data = subset(cr,exposure=="NDVI"&outcome=="LBW"))+
   geom_ribbon(aes(x=x,y=y,ymin=ylow,ymax=yhigh),
                     fill="#002C5B",alpha=0.4)+
   geom_line(aes(x=x,y=y),colour="#002C5B88",size=0.5)+
   facet_grid(~outcome,scales = "free",
                    labeller = labeller(exposure=label_parsed))+
   annotate("text",x=0.2,y=0.85,size=4,color="grey20",hjust=0,
                 label=expression("Nonlinear "*italic(P)*" < 0.001"))+
   scale_x_continuous(breaks = seq(0,0.8,0.2),
                               labels = seq(0,0.8,0.2))+
   scale_y_continuous(breaks=seq(0.5,1,by=0.1),
                               labels=sprintf("%.1f",seq(0.5,1,0.1)))+
   labs(title = "a",y="OR (95% CI)",x="")+
   theme_classic()+theme0+
   theme(axis.text.x = element_blank(),
            strip.background.x = element_rect(color = "white",
                                                         fill = "#002C5B40"),
            plot.margin = margin(b=-0.2,t=0.2,r=0.2,l=0.2,
                                           unit = "cm"))->p1
ggplot()+
   geom_boxplot(aes(x=data$NDVI3000,y=-0.2),width=0.24,
                      fill="#002C5B44",color="#002C5B88",
                      outlier.shape = NA)+
   geom_density(aes(x=data$NDVI3000),fill="#002C5B66",
                      color="white",show.legend = F)+
   scale_x_continuous(breaks = seq(0,0.8,0.2),
                               labels = seq(0,0.8,0.2))+
   labs(x=expression("NDVI"["-3000m"]),y="Kernel density (%)")+
   theme_classic()+theme0+
   theme(plot.margin = margin(t=-0.35,b=0,l=0.2,r=0.2,
                                     unit = "cm"))->p2
#############NDVI+TLBW
ggplot(data = subset(cr,exposure=="NDVI"&outcome=="TLBW"))+
   geom_ribbon(aes(x=x,y=y,ymin=ylow,ymax=yhigh),
                     fill="#B30839",alpha=0.4)+
   geom_line(aes(x=x,y=y),colour="#B3083988",size=0.5)+
   facet_grid(~outcome,scales = "free",
                    labeller = labeller(exposure=label_parsed))+
   annotate("text",x=0.2,y=0.85,size=4,color="grey20",hjust=0,
                 label=expression("Nonlinear "*italic(P)*" < 0.001"))+
   scale_x_continuous(breaks = seq(0,0.8,0.2), labels = seq(0,0.8,0.2))+
```

```
  scale_y_continuous(breaks=seq(0.5,1,by=0.1),
                     labels=sprintf("%.1f",seq(0.5,1,0.1)))+
  labs(title = "",y=NULL,x="")+
  theme_classic()+theme0+
  theme(axis.text.x = element_blank(),
        strip.background.x = element_rect(color = "white",
                                          fill = "#B3083940"),
        plot.margin = margin(b=-0.2,t=0.2,r=0.2,l=0.2,
                             unit = "cm"))->p3
ggplot()+
  geom_boxplot(aes(x=data$NDVI3000,y=-0.2),width=0.24,
               fill="#B3083944",color="#B3083988",
               outlier.shape = NA)+
  geom_density(aes(x=data$NDVI3000),fill="#B3083966",
               color="white",show.legend = F)+
  scale_x_continuous(breaks = seq(0,0.8,0.2),
                     labels = seq(0,0.8,0.2))+
  labs(x=expression("NDVI"["-3000m"]),y=NULL)+
  theme_classic()+theme0+
  theme(plot.margin = margin(t=-0.35,b=0,l=0.2,r=0.2,
                             unit = "cm"))->p4

#############EVI+LBW
ggplot(data = subset(cr,exposure=="EVI"&outcome=="LBW"))+
  geom_ribbon(aes(x=x,y=y,ymin=ylow,ymax=yhigh),
              fill="#002C5B",alpha=0.4)+
  geom_line(aes(x=x,y=y),colour="#002C5B88",size=0.5)+
  facet_grid(~outcome,scales = "free",
             labeller = labeller(exposure=label_parsed))+
  annotate("text",x=0.2,y=0.85,size=4,color="grey20",hjust=0,
           label=expression("Nonlinear "*italic(P)*" < 0.001"))+
  scale_x_continuous(breaks = seq(0.1,0.7,0.2),
                     labels = seq(0.1,0.7,0.2))+
  scale_y_continuous(breaks=seq(0.5,1,by=0.1),
                     labels=sprintf("%.1f",seq(0.5,1,0.1)))+
  labs(title = "b",y="OR (95% CI)",x="")+
  theme_classic()+theme0+
  theme(axis.text.x = element_blank(),
        strip.background.x = element_rect(color = "white",
                                          fill = "#002C5B40"),
        plot.margin = margin(b=-0.2,t=0.2,r=0.2,l=0.2,
                             unit = "cm"))->p5
```

```
ggplot()+
  geom_boxplot(aes(x=data$EVI3000,y=-0.28),width=0.32,
               fill="#002C5B44",color="#002C5B88",
               outlier.shape = NA)+
  geom_density(aes(x=data$EVI3000),fill="#002C5B66",
               color="white",show.legend = F)+
  scale_x_continuous(breaks = seq(0.1,0.7,0.2),
                     labels = seq(0.1,0.7,0.2))+
  scale_y_continuous(breaks = seq(0,4,1),
                     labels = seq(0,4,1))+
  labs(x=expression("EVI"["-3000m"]),
       y="Kernel density (%)")+
  theme_classic()+theme0+
  theme(plot.margin = margin(t=-0.35,b=0,l=0.2,r=0.2,
                             unit = "cm"))->p6
#############EVI+TLBW
ggplot(data = subset(cr,exposure=="EVI"&outcome=="TLBW"))+
  geom_ribbon(aes(x=x,y=y,ymin=ylow,ymax=yhigh),
              fill="#B30839",alpha=0.4)+
  geom_line(aes(x=x,y=y),colour="#B3083988",size=0.5)+
  facet_grid(~outcome,scales = "free",
             labeller = labeller(exposure=label_parsed))+
  annotate("text",x=0.2,y=0.85,size=4,color="grey20",hjust=0,
           label=expression("Nonlinear "*italic(P)*" < 0.001"))+
  scale_x_continuous(breaks = seq(0.1,0.7,0.2),
                     labels = seq(0.1,0.7,0.2))+
  scale_y_continuous(breaks=seq(0.5,1,by=0.1),
                     labels=sprintf("%.1f",seq(0.5,1,0.1)))+
  labs(title = "",y=NULL,x="")+
  theme_classic()+theme0+
  theme(axis.text.x = element_blank(),
        strip.background.x = element_rect(color = "white",
                                          fill = "#B3083940"),
        plot.margin = margin(b=-0.2,t=0.2,r=0.2,l=0.2,
                             unit = "cm"))->p7
ggplot()+
  geom_boxplot(aes(x=data$EVI3000,y=-0.28),width=0.32,
               fill="#B3083944",color="#B3083988",
               outlier.shape = NA)+
  geom_density(aes(x=data$EVI3000),fill="#B3083966",
               color="white",show.legend = F)+
  scale_x_continuous(breaks = seq(0.1,0.7,0.2),
                     labels = seq(0.1,0.7,0.2))+
```

```
    scale_y_continuous(breaks = seq(0,4,1),
                                   labels = seq(0,4,1))+
    labs(x=expression("EVI"["-3000m"]),y=NULL)+
    theme_classic()+theme0+
    theme(plot.margin = margin(t=-0.35,b=0,l=0.2,r=0.2,
                                         unit = "cm"))->p8
#############
library(ggpubr)
aa<-ggarrange(p1,p2,ncol = 1,align = "v",heights = c(2,1))
bb<-ggarrange(p3,p4,ncol = 1,align = "v",heights = c(2,1))
cc<-ggarrange(p5,p6,ncol = 1,align = "v",heights = c(2,1))
dd<-ggarrange(p7,p8,ncol = 1,align = "v",heights = c(2,1))
p<-ggarrange(aa,bb,cc,dd,
             nrow = 1,ncol = 4,align = "v")
cairo_pdf("figure3.pdf",width = 11,height = 4,family = "Calibri")
p
dev.off()
```

4. Codes for Figure 4

```
library(xlsx);library(dplyr);library(ggsci);library(ggplot2)
sub<-read.xlsx("Results.xlsx",sheetIndex = 3)
##############################
sub$outcome <- factor(sub$outcome,levels = c("LBW","TLBW"),
                                 labels=c("LBW","TLBW"))
mutate(sub,y=round(exp(beta)^0.1,3),
       ylow=round(exp(low)^0.1,3),
       yhigh=round(exp(high)^0.1,3))->sub
sub$exposure <- factor(sub$exposure,levels = c("NDVI","EVI"),
                              labels=c(expression("NDVI"["-3000m"]),expression("EVI"["-3000m"])))
sub$group <- c(rep(c("≤25","(25, 35]",">35"),4),
                  rep(c("Below high school","High school",
                        "College or above"),4),
                  rep(c("City","Village"),4))
sub$group <- factor(sub$group,levels = c("≤25","(25, 35]",">35","Below high school",
                                          "High school","College or above","City","Village"))
color1 <- c(rep(c('#b5e1f7','#fd8a09','#ca2c32'),4),
            rep(c('#b5e1f7','#fd8a09','#ca2c32'),4),
            rep(c('#b5e1f7','#fd8a09'),4))
p <- ggplot()+geom_pointrange(data=sub,size=0.45,
                              aes(x=group,y=y, ymin = as.numeric(ylow),
                                  ymax = as.numeric(yhigh)),shape=15,width=1.5)+
```

```
  geom_errorbar(data=sub,size=0.45,
                aes(x=group,y=y,
                    ymin = as.numeric(ylow),
                    ymax = as.numeric(yhigh)),width=0.15)+
  geom_point(data = sub,size=3,
             aes(x=group,y=y),shape=21,colour="black",fill=color1)+
  facet_grid(exposure~outcome,scales="free",labeller="label_parsed")+
  geom_vline(xintercept = "High school",lwd=49,col="grey50",alpha=0.1)+
  geom_vline(xintercept = 7.55,lwd=33,col="black",alpha=0.1)+
  #geom_hline(yintercept = )
  scale_fill_npg()+
  ylab("OR (95% CI)")+
  theme_test()+
  theme(panel.spacing.x = unit(0.2,"cm"),
        panel.spacing.y = unit(0.1, "cm"),
        axis.title = element_blank(),
        axis.text = element_text(color="black"),
        axis.text.x=element_text(size=12,angle = 45,vjust=1,hjust=1),
        axis.ticks.x=element_blank(),
        axis.text.y = element_text(size = 12),
        axis.title.y.left=element_text(size=14,color="black"),
        legend.text = element_text(color="black",size=9),
        legend.title=element_blank(),
        legend.spacing.x=unit(0.1,'cm'),
        legend.key=element_blank(),
        legend.key.width=unit(0.4,'cm'),
        legend.key.height=unit(0.4,'cm'),
        legend.position = "top",
        strip.background = element_rect(fill = "white",colour = "black",size=0.3),#3182bd
#fill =gray(0.85),colour = gray(0.3),
        strip.text.y=element_text(size=14,face = "bold"),
        strip.text.x=element_text(size=14,face = "bold"),
        plot.margin=unit(c(0.3,0.3,0.3,0.3),units=,"cm"))
cairo_pdf("figure4.pdf",width=9,height=8,family = "Calibri")
p
dev.off()
```

5. Codes for Figure 5

```
library(xlsx);library(ggplot2);library(ggthemes)
data<-read.xlsx("Results.xlsx",sheetIndex = 4)
p1 <- ggplot(data,aes(x=exposure,y=af*100,fill=outcome))+
  geom_bar(stat = 'identity',position = position_dodge(0.75), width=0.75,color="black")+
```

```
  geom_text(aes(label=round(af*100,1)),size=4,vjust=3,
            position = position_dodge(0.75),color="black")+
  geom_vline(xintercept = 6.6,lwd=142,col="grey50",alpha=0.1)+
  geom_errorbar(aes(x=exposure,ymin=aflow*100, ymax=afhigh*100),
                stat = 'identity', position=position_dodge(.75), width=.2) +
  scale_fill_manual(values = c("#b3cde3","#fbb4ae"))+
  scale_y_continuous(limits = c(0,8),breaks = seq(0,8,2), labels = seq(0,8,2))+
  scale_x_discrete(limits=c(paste0("NDVI",c(500,1000,2000,3000)),
                            paste0("EVI",c(500,1000,2000,3000))),
                   labels=c(expression(NDVI["-500m"]),expression(NDVI["-1000m"]),
                            expression(NDVI["-2000m"]),expression(NDVI["-3000m"]),
                            expression(EVI["-500m"]),expression(EVI["-1000m"]),
                            expression(EVI["-2000"]),expression(EVI["-3000m"])))+
labs(title = "a",y="Attributable fractions (%)",x=NULL)+
  theme_calc(base_size = 12,base_family = "Calibri")+
  theme(axis.text.x = element_text(size = 12),
        axis.text.y = element_text(size = 12),
        axis.title.y = element_text(size = 14),
        legend.position = c(0.1,0.89),
        legend.direction = "horizontal",
        legend.background = element_blank(),
        legend.title = element_blank(),
        plot.title = element_text(size = 16,face = "bold"),
        plot.background = element_blank()
  )
##
p2 <- ggplot(data,aes(x=exposure,y=an/1000,fill=outcome))+
  geom_bar(stat = 'identity',position = position_dodge(0.75),width=0.75,color="black")+
  geom_text(aes(label=format(round(an,0),0)),size=4,vjust=3,
            position = position_dodge(0.75),color="black")+
  geom_vline(xintercept = 6.6,lwd=142,col="grey50",alpha=0.1)+
  geom_errorbar(aes(x=exposure,ymin=low, ymax=high),
                stat = 'identity',
                position=position_dodge(.8), width=.2) +
  scale_fill_manual(values = c("#b3cde3","#fbb4ae"))+
  scale_y_continuous(limits = c(0,6000),breaks = seq(0,6000,2000),
                     labels = seq(0,6000,2000))+
  scale_x_discrete(limits=c(paste0("NDVI",c(500,1000,2000,3000)),
                            paste0("EVI",c(500,1000,2000,3000))),
                   labels=c(expression(NDVI["-500m"]),expression(NDVI["-1000m"]),
                            expression(NDVI["-2000m"]),expression(NDVI["-3000m"]),
                            expression(EVI["-500m"]),expression(EVI["-1000m"]),
                            expression(EVI["-2000"]),expression(EVI["-3000m"])))+
```

```
    labs(title = "b",y= "Avoidable number",x=NULL)+
    theme_calc(base_size = 12,base_family = "Calibri")+
    theme(axis.text.x = element_text(size = 12),
            axis.text.y = element_text(size = 12),
            axis.title.y = element_text(size = 14),
            legend.position = c(0.1,0.89),
            legend.direction = "horizontal",
            legend.background = element_blank(),
            legend.title = element_blank(),
            plot.title = element_text(size = 16,face = "bold"),
            plot.background = element_blank())
##
library(ggpubr)
p<-ggarrange(p1,p2,ncol = 1,align = "v")
cairo_pdf("figure/figure5.pdf",width = 9,height = 8,family = "Calibri")
p
dev.off()
```
