# Supplementary material for: Surrounding greenness is associated with lower risk and burden of low birth weight in Iran
Source: Nat Commun. 2023 Nov 21;14:7595. doi: 10.1038/s41467-023-43425-6 (PMC10663448; doi:10.1038/s41467-023-43425-6)
Supplement: Supplementary file 5 — Reporting Summary [file 41467_2023_43425_MOESM5_ESM.pdf]

nature portfolio

Corresponding author(s): YunquanZhang; Fatemeh Mayvaneh

Last updated by author(s): Oct 25, 2023

Reporting Summary

Nature Portfolio wishes to improve the reproducibility of the work that we publish. This form provides structure for consistency and transparency in reporting. For further information on Nature Portfolio policies, see our Editorial Policies and the Editorial Policy Checklist.

Statistics

For all statistical analyses, confirm that the following items are present in the figure legend, table legend, main text, or Methods section.

n/a	Confirmed	
☒	☐	The exact sample size (*n*) for each experimental group/condition, given as a discrete number and unit of measurement
☒	☐	A statement on whether measurements were taken from distinct samples or whether the same sample was measured repeatedly
☐	☒	The statistical test(s) used AND whether they are one- or two-sided<br>*Only common tests should be described solely by name; describe more complex techniques in the Methods section.*
☐	☒	A description of all covariates tested
☒	☐	A description of any assumptions or corrections, such as tests of normality and adjustment for multiple comparisons
☐	☒	A full description of the statistical parameters including central tendency (e.g. means) or other basic estimates (e.g. regression coefficient) AND variation (e.g. standard deviation) or associated estimates of uncertainty (e.g. confidence intervals)
☐	☒	For null hypothesis testing, the test statistic (e.g. *F*, *t*, *r*) with confidence intervals, effect sizes, degrees of freedom and *P* value noted<br>*Give P values as exact values whenever suitable.*
☒	☐	For Bayesian analysis, information on the choice of priors and Markov chain Monte Carlo settings
☒	☐	For hierarchical and complex designs, identification of the appropriate level for tests and full reporting of outcomes
☒	☐	Estimates of effect sizes (e.g. Cohen's *d*, Pearson's *r*), indicating how they were calculated

Our web collection on statistics for biologists contains articles on many of the points above.

Software and code

Policy information about availability of computer code

Data collection	Data used in this study are directly downloaded, and corresponding sources have been shown in the following Data section.
Data analysis	All the analyses were performed in R software (version 4.0.2). The analytic and drawing codes are provided in the supplementary materials.

For manuscripts utilizing custom algorithms or software that are central to the research but not yet described in published literature, software must be made available to editors and reviewers. We strongly encourage code deposition in a community repository (e.g. GitHub). See the Nature Portfolio guidelines for submitting code & software for further information.

Data

Policy information about availability of data

All manuscripts must include a data availability statement. This statement should provide the following information, where applicable:

- Accession codes, unique identifiers, or web links for publicly available datasets
- A description of any restrictions on data availability
- For clinical datasets or third party data, please ensure that the statement adheres to our policy

Satellite-derived datasets for NDVI and EVI are accessed at https://modis.gsfc.nasa.gov/data/dataprod/mod13.php. Meteorological factors are available at https://cds.climate.copernicus.eu/cdsapp#!/dataset/10.24381/cds.6c68c9bb?tab=overview. Ground-level estimates of PM2.5 are obtained from https://sites.wustl.edu/acag/datasets/surface-pm2-5/. Gridded birth population data for Iran are derived from https://hub.worldpop.org/geodata/listing?id=18. Provincial-level SES

variables are collected from https://www.amar.org.ir/english/Iran-Statistical-Yearbook. The raw birth cohort data are secret due to local regulations and privacy laws, but are available from the corresponding authors upon reasonable request.

Research involving human participants, their data, or biological material

Policy information about studies with human participants or human data. See also policy information about sex, gender (identity/presentation), and sexual orientation and race, ethnicity and racism.

Reporting on sex and gender	Among 4,021,741 live births, females accounted for 48.4%. Neonatal sex was adjusted in the models based on prior literatures.
Reporting on race, ethnicity, or other socially relevant groupings	In this study, we divided pregnant women into Iranian and non-Iranian according to their nationality. Nationality is adjusted for all analyses to account for different susceptibility in exposure and outcome.
Population characteristics	Among 4,021,741 live births in our study, 263,728 (6.6%) were LBW and 121,852 (3.0%) were TLBW. The average (± standard deviation, SD) birth weight of LBW and TLBW infants were 2024.8 ± 479.2 g and 2196.7 ± 407.9 g, respectively. About a quarter of mothers with LBW/TLBW infants were village residents, and nearly a half were less than 25 or above 35 years old and below high school education.
Recruitment	Not applicable
Ethics oversight	The research proposal was granted by the Ethics Committee of Sabzevar University of Medical Sciences (IR.MEDSAB.REC.1396.99). All participants provided written informed consent.

Note that full information on the approval of the study protocol must also be provided in the manuscript.

Field-specific reporting

Please select the one below that is the best fit for your research. If you are not sure, read the appropriate sections before making your selection.

☐ Life sciences ☐ Behavioural & social sciences ☒ Ecological, evolutionary & environmental sciences

For a reference copy of the document with all sections, see nature.com/documents/nr-reporting-summary-flat.pdf

Ecological, evolutionary & environmental sciences study design

All studies must disclose on these points even when the disclosure is negative.

Study description	By incorporating over 4 million mother-infant pairs, we conducted logistic regression models to quantify the association of greenness exposures with LBW/TLBW, and derived exposure-response relationships between greenness exposure during pregnancy and risk of LBW/TLBW in Iran. Under predefined causality and counterfactual scenarios of improved NDVI or EVI, we estimated greenness-related attributable fractions and avoidable numbers of LBW/TLBW by linking the exposure-response functions to spatially resolved estimates of live births, and maternal greenness exposures in 2015.
Research sample	A total of 4,068,843 mother-infant pairs during from 2013 to 2018 were collected from Neonatal Health Office of the Iranian Ministry of Health and Medical Education. All eligible records were included in this study.
Sampling strategy	All eligible records registered with Neonatal Health Office of the Iranian Ministry of Health and Medical Education were included in the analysis. These records were from 749 hospitals in 31 provinces in Iran, which could represent Iranian newborns well. No further sampling procedure was conducted.
Data collection	Birth records occurring in 749 hospitals across 31 Iranian provinces from January 2013 to December 2018 were collected from Ministry of Health and Medical Education, who owns and operates the largest health care delivery network in Iran. Detailed information for gravidas and neonates were extracted from electronic archives, which were established and managed by obstetricians.
Timing and spatial scale	We collected all birth records registered with Neonatal Health Office of the Iranian Ministry of Health and Medical Education from January 2013 to December 2018. These birth data were from 749 hospitals covering 31 provinces across Iran.
Data exclusions	To assess the association between low birth weight and vegetation indices, we excluded stillbirths, neonatal deaths and records with undetermined sex and unmatched exposure.
Reproducibility	All attempts to repeat the analysis were successful.
Randomization	This analysis was an observational study and there were no experimental groups.
Blinding	Blinding was not relevant to this study, as it was an observational study using birth records from hospitals.

Did the study involve field work? ☐ Yes ☒ No

Reporting for specific materials, systems and methods

We require information from authors about some types of materials, experimental systems and methods used in many studies. Here, indicate whether each material, system or method listed is relevant to your study. If you are not sure if a list item applies to your research, read the appropriate section before selecting a response.

Materials & experimental systems

n/a	Involved in the study
☒	☐ Antibodies
☒	☐ Eukaryotic cell lines
☒	☐ Palaeontology and archaeology
☒	☐ Animals and other organisms
☒	☐ Clinical data
☒	☐ Dual use research of concern
☒	☐ Plants

Methods

n/a	Involved in the study
☒	☐ ChIP-seq
☒	☐ Flow cytometry
☒	☐ MRI-based neuroimaging
